# Theta burst stimulation add on to dialectical behavioral therapy in borderline-personality-disorder: methods and design of a randomized, single-blind, placebo-controlled pilot trial

**DOI:** 10.1007/s00406-023-01692-w

**Published:** 2023-09-15

**Authors:** Milenko Kujovic, Daniel Benz, Mathias Riesbeck, Christian Bahr, Christian Kriegs, Dirk Reinermann, Michaela Jänner, Susanne Neufang, Zsofia Margittai, Daniel Kamp, Christian Plewnia, Eva Meisenzahl

**Affiliations:** 1 Department of Psychiatry and Psychotherapy, Medical Faculty, Heinrich-Heine University Düsseldorf, Düsseldorf, Germany; 2 Department of Psychiatry and Psychotherapy, University Hospital of Psychiatry and Psychotherapy, Tübingen, Germany

**Keywords:** DBT, rTMS, BPD, Randomized trial, Theta burst stimulation

## Abstract

Specialized psychotherapeutic treatments like dialectical behavioral therapy (DBT) are recommended as first treatment for borderline personality disorder (BPD). In recent years, studies have emerged that focus on repetitive transcranial magnetic stimulation (rTMS) in BPD. Both have independently demonstrated efficacy in the treatment of BPD. Intermitted theta burst stimulation (iTBS), a modified design of rTMS, is thought to increase the excitability of neurons and could be a supplement to psychotherapy in addition to being a standalone treatment. However, no studies to date have investigated the combination of DBT and rTMS/iTBS. This study protocol describes the methods and design of a randomized, single-blinded, sham-controlled clinical pilot study in which BPD patients will be randomly assigned to either iTBS or sham during four consecutive weeks (20 sessions in total) in addition to standardized DBT treatment. The stimulation will focus on the unilateral stimulation of the left dorsolateral prefrontal cortex (DLPFC), which plays an important role in the control of impulsivity and risk-taking. Primary outcome is the difference in borderline symptomatology, while secondary target criteria are depressive symptoms, general functional level, impulsivity and self-compassion. Statistical analysis of therapy response will be conducted by Mixed Model Repeated Measurement using a 2 × 2-factorial between-subjects design with the between-subject factor stimulation (TMS vs. Sham) and the within-subject factor time (T0 vs. T1). Furthermore, structural magnetic resonance imaging (MRI) will be conducted and analyzed. The study will provide evidence and insight on whether iTBS has an enhancing effect as add-on to DBT in BPD.

*Trial registration*: drks.de (DRKS00020413) registered 13/01/2020.

## Background

Borderline Personality Disorder (BPD) is a debilitating condition characterized by a pervasive pattern of difficulty in regulating emotions, impulsivity, instability of self-image and self-harming behavior often resulting in significant interpersonal problems and impaired quality of life. Its prevalence is estimated to be around 1–2% [1]. Moreover, BPD has high rates of comorbidities, with being highest for mood disorders [2].

A number of meta-analyses investigating the neurobiological underpinnings of BPD have reliably demonstrated an impaired fronto-limbic brain network, with hyperactivity in limbic and hypoactivity in frontal regions [3–5], resulting in diminished top-down inhibitory control and a corresponding difficulty in regulating emotions and impulsive behavior.

Attempts have been made to develop appropriate psychotherapy programs to alleviate the symptoms of BPD. The most wide-spread, evidence based approach is dialectic behavioral therapy (DBT) [6]. The essential goal of DBT is to enable patients with BPD to learn how to regulate their emotions through the implementation of distress tolerance, mindfulness and acceptance and thus to experience improved quality of life, reduced self-harming and suicidal behaviors, a more stable self-image and interpersonal relationships.

Evidence has started to emerge showing that treatment with DBT also results in changes in neural activation that correspond to improvements in emotion regulation in patients with BPD. A pilot study by Schnell and Herpertz [7] examined patients with BPD receiving a 12-week in-patient DBT program using fMRI at five time points during treatment and found evidence of amygdala function normalization in the course of therapy. Goodman et al. [8] provided evidence for improved amygdala habituation to repeated unpleasant images in patients with BPD after 12-month outpatient DBT, which was associated with enhanced emotion regulation measures. In addition to this, Mancke et al. [9] found an increased grey matter volume in cortical regions connected with emotion regulation in women with BPD after receiving DBT treatment. These findings are supported by the review conducted by Iskric and Barkley-Levenson [10]. However, current published findings on the neural correlates of DBT effects are still scarce and much further research is needed to draw definitive conclusions.

Repetitive transcranial magnetic stimulation (rTMS) has emerged as a treatment option for different psychiatric disorders in recent years [11]. Though most studies have examined its efficacy in treating depression, initial attempts have also been made to apply it to other psychiatric disorders such as post-traumatic stress disorder, eating disorders and obsessive–compulsive disorder [12]. The efficacy of rTMS for the treatment of depression has now been confirmed in several randomized controlled trials and meta-analyses [13, 14], while its role in the therapy of other disorders still requires further research. For the treatment of BPD its application has been investigated in several studies and estimated in one meta-analysis by Konstantinou et al. [15]. Gündoğmuş et al. [16] reported a reduction in impulsivity, one of the BPD core symptoms, after rTMS in a case report which was later supported in a randomized trial by Lisoni et al. [17]. Overall, a reduction of symptom severity after rTMS stimulation was reported [18] and Rachid [19] emphasized that TMS were safe and potentially effective in reduction of symptoms of BPD.

One experimental investigation shows that the temporary disruption of the left lateral prefrontal cortex function using rTMS in healthy participants leads to increased impulsive behavior and decreased self-control regulatory processes [20]. Emotion in healthy subjects is modified by the cognitive control processes originating in the dorsolateral prefrontal cortex (DLPFC) [21]. This part of the prefrontal cortex processes voluntary emotion regulation, which includes suppression of emotional expression, selective attention, overcoming interference from emotional distractors, inhibition of emotional motor response and reappraisal. In addition, DLPFC is involved in balancing the value of emotions and regulation of the valence of emotional experiences as one of higher cognitive functions [21, 22]. While the right DLPFC is reported to be reflective and responsible for synthesis, the left DLPFC is associated with the control of impulsivity and risk-taking, and responsible for analysis [23]. Most widely used in the treatment of major depressive disorder is a high-frequency rTMS over the left DLPFC [24]. Furthermore, as mentioned above, patients with BPD have a dysfunction in fronto-limbic functional connectivity and hypoactivity in frontal region as an anatomical correlate of the disorder [25, 26]. Thus, the goal of both DBT and rTMS is to ameliorate this imbalance, which is expected to lead to less impulsive behavior and increased top-down inhibitory control in borderline sufferers.

Theta-burst stimulation (TBS) is an advanced TMS-protocol, aiming for optimized long-term potentiation (LTP) by mimicking Theta-rhythm (5–10 Hz), which is observed in mammalian brain during mnemonic processing, originally discovered in hippocampal pyramidal neurons, and considered to be critical in LTP [27]. Furthermore, brief, high-frequency-pulses at about 50–100 Hz are proven to induce intracellular signaling associated with LTP. Accordingly, TBS-stimulation schemes consist of short high-frequency bursts of 100 Hz, 3–5 pulses each, applied in 5 Hz frequency. Continuous bursts (cTBS) diminishes and intermittent bursts (iTBS) enhance the excitability of neurons in the targeted area [28, 29]. Furthermore, iTBS can be used to treat psychiatric disorders with a comparable effectiveness, but through shorter session duration [30].

A novel idea in terms of brain stimulation methods is to use rTMS or iTBS as a supplement to psychotherapy in addition to being a standalone treatment. For instance, iTBS has been shown to increase the effectiveness of cognitive behavioral therapy for smoking cessation [31]. Investigating the idea of combining non-invasive brain stimulation techniques with psychotherapy approaches is important, as it potentially decreases the rate of non-response in any monotherapy, which is still relatively high for several psychiatric disorders [32].

Although findings exist that prove the efficacy of DBT and rTMS in treating borderline symptoms as separate treatments as mentioned above, there is no study that investigated the effect of both treatments combined. In addition, iTBS allows for a shorter session duration than traditional rTMS protocols, which is expected to be better tolerated by impulsive patients, thus being beneficial in terms of compliance. Thus, we conducted a randomized single-blind study to investigate whether an add-on iTBS improves efficacy of DBT in patients with BPD and comorbid major depression receiving standardized inpatient therapy. We hypothesize that unilateral stimulation of the left DLPFC, which plays a role in impulsivity and risk-taking [23], and is the most common target in treatment of major depression [24], combined with DBT will result in a higher reduction of borderline symptoms (by means of improved emotion regulation skills in a combination with reduced impulsivity as well as improved depressive symptoms) compared to DBT plus placebo.

## Methods

### Procedure

**Fig. 1:**
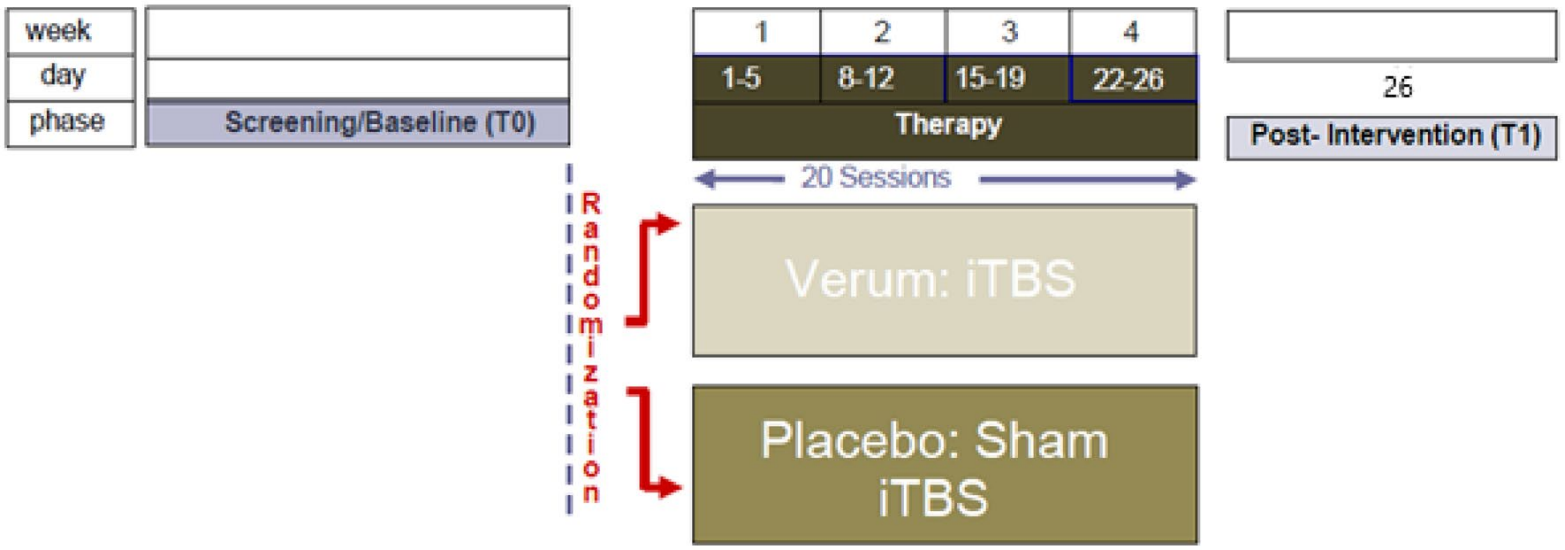
Intervention scheme/time course

Patients with BPD undergoing an 8–12-week routine inpatient DBT treatment in one specialized ward of a German psychiatric hospital are screened for eligibility after 4 weeks of DBT-treatment. After reaching informed consent they are additionally randomly allocated (1:1) to receive 20 sessions iTBS (DBT + iTBS) or 20 sessions placebo/sham stimulation (DBT + sham; each 1 per day from Monday to Friday) from the 5th to the 8th week of the treatment (see Fig. 1). Randomization procedure is based on an algorithm created with the programming platform MATLAB. Patients are blind regarding intervention (iTBS) or placebo (sham) whereas study personnel is not. Furthermore, data analyses will also be done blinded regarding group allocation.

The study protocol was finished in July 2019 and the trial was registered at DRKS in January 2020. Screening and inclusion of patients started in January 2021. All included patients sign the written declaration of consent, patient information and information on data protection. The study was approved by the Ethics Committee of the medical faculty of the Heinrich Heine University, Duesseldorf.

Routine DBT-treatment is provided based on an adaptation for inpatient treatment in Germany by Bohus et al. [33]. The structured, modularized program contains the modules skills training, interpersonal skills, dealing with feelings, and mindfulness. It is conducted in weekly group sessions including psychoeducation and training elements and additional one to two individual sessions per week. Psychoeducation is aimed at explaining BPD to affected individuals. This includes theories of how the disorder arises, comorbidities and ways to lead a healthy life despite persisting symptoms. Additionally, concepts like the dialectical view on the disorder and the importance of sleep hygiene are taught. Regarding DBT skills, the module aims at teaching simple models of emotion, including strategies on how to control them. Additionally, patients experience ways to deal with interpersonal problems as well as skills to tolerate distress caused by crisis leading to, e.g., self-harm pressure or severe suicidal ideation. The module DBT tools teaches a deeper understanding of oneself, including behavior analysis, problem solving as well as instrumental, primary and secondary emotions. Furthermore, it aims at creating self-compassion in participants. The therapeutic team consists of either fully trained psychotherapists or psychologists in training. The therapist in charge is DBT-certified. Lastly, patients receive psychiatric care by an individually assigned nurse once a week.

### Inclusion/exclusion criteria

Patients between 18 and 45 years of age and diagnosed with a borderline personality disorder and comorbid Major Depression are eligible for our study. Diagnoses are assessed using the standardized clinical interviews, Diagnostisches Kurzinterview bei psychischen Störungen (Mini-DIPS OA; [34]) and the Structured Clinical Interview for DSM-5 Personality Disorders (SCID-V-PD; [35]). All patients participate in an in-patient standardized DBT treatment in our clinic. Patients had to have no previous knowledge of DBT. They need to have a sufficient knowledge of the German language and be able to give informed consent. In case of drug treatment, stable intake in therapeutic doses for two weeks before the start of the study stimulation phase, is necessary, remaining stable during the 4-weeks stimulation phase. For female patients, further inclusion criteria includes negative pregnancy test and willingness to use contraception for the duration of the stimulation. Exclusion criteria are: history of seizures (epilepsy), metallic foreign objects in the skull, pronounced tattoos in the region of the head, significant brain malformations or tumors, cerebral-vascular events, traumatic brain injuries, neurodegenerative diseases, brain surgery, deep brain stimulation, other intracranial implants, cardiac pacemaker, other serious physical illness and other psychiatric comorbidities beside major depression and borderline personality disorder. Moreover, patients with acute suicidality (MADRS score > 4 for question 10), tinnitus, pregnancy, claustrophobia, current or previous treatment with electroconvulsive therapy or vagus nerve stimulation are not eligible. Patients taking anti-epileptic medication including benzodiazepines at a dose equal to or greater than 1 mg/day of lorazepam are also excluded as well as, patients under legal guardianship with reservation of consent.

Before study enrollment, all patients will be scanned to exclude alterations in brain morphology. In case of more than four missed iTBS/sham sessions patients will also be excluded from the analyses. However, all other patients will be included irrespective of later dropout.

### iTBS treatment

The stimulation is applied with a PowerMAG Research 100 magnetic stimulator [36] and a PMD70-pCool figure of eight coil. The devices have the European Certification (CE) mark and are used exclusively for the purpose specified by the manufacturer and are only operated, used and maintained by people who have the necessary training, knowledge and experience. The resting motor threshold will be determined automatically using electromyography before the first treatment and two weeks later, with the integration of the motor evoked potential and the algorithmic determination of the threshold [37–39]. The stimulation targets the left DLPFC, which will be located using the Beam-F3 method [40]. Each treatment session with iTBS consists of 600 stimuli. The intervention will be applied at 80% of the resting motor threshold intensity. The stimulation is administered intermittently (two seconds stimulation, eight seconds pause), the duration of a treatment unit will be three minutes and twelve seconds. The sham stimulation is carried out with a double coil PMD70-pCool-Sham [41]. The PMD70-pCool-Sham coil's reduced magnetic field strength allows for stimulation of only the nearby scalp, producing a twitching sensation without affecting the brain, and it has equivalent weight and sound to the active coil [41]. Subjects in the sham group experience noises and physical sensations similar to the verum stimulation.

### Assessments

After successful study enrollment, before commencement of the stimulation, the following target parameters are recorded at baseline (see Table 1).

**Table 1:** Assessment schedule throughout the study

Time	Screen-ing		Baseline	Treatment phase																				Post-intervention examination
Week				1					2					3					4					
Visit	T0			V1	V2	V3	V4	V5	V 8	V9	V10	V11	V 12	V 15	V 16	V 17	V 18	V 19	V22	V23	V24	V25	V26	T1
Day			− 3	1	2	3	4	5	8	9	10	11	12	15	16	17	18	19	22	23	24	25	26	26
MRI	X																							
Consent/information/data protection	X																							
Inclusion/exclusion criteria	X																							
SKID V PD	X																							
Mini-DIPS-OA	X																							
Psychiatric history	X																							
Medical history	X																							
Physical examination	X																							
Neurological Examination	X																							
MADRS	X												X											X
Pregnancy test	X																							
BSL-23			X					X					X					X						X
GAF			X																					X
BIS-15			X																					X
BDI-II			X					X					X					X						X
SCS-D			X																					X
Default motor threshold				X										X										

*Note*. T0 = baseline, V1–V26 = treatment phase, T1 = post-treatment

Short version of Borderline Symptom List (BSL-23) [42], second edition of Beck’s Depression Inventory (BDI-II) [43], Global Assessment of Functioning Scale (GAF) [44] and the Baratt Impulsiveness Scale, short form (BIS-15) [45]. Further, the German short version of the Self Compassion Scale (SCS-D) [46], the Montgomery–Åsberg Depression Rating Scale (MADRS) [47] and a Delay Discounting task [48] will be used (see detailed description below).

At the end of each week with iTBS/sham intervention, patients are asked to complete a BSL-23 and BDI-II questionnaire to assess symptom severity. Possible undesirable side effects of the stimulation are assessed and recorded by the practitioner after each treatment according to routine trial (serious) adverse events monitoring. At the end of the intervention period (after four weeks), further assessments are made for MADRS, GAF, BIS-15, SCS-D. Moreover, to check for a potential experimenter bias, participants’ belief about whether they took part in the verum or sham treatment condition are assessed after the first iTBS session and at post-intervention.

### Primary outcome measures

Primary outcome is the difference of improvement of borderline symptomatology between the two groups quantified by changes in the short version of the BSL-23 [42, 49] from baseline (T0) to post intervention (T1). The BSL-23 is a validated self-rating measure of borderline personality disorder symptoms. It contains 23 items that are scored from 0 = not at all to 4 = very strong. All items concern the last week, e.g. ‘In the course of last week, I felt helpless’ or ‘In the course of last week I was afraid of losing control’. The BSL-23 has shown good convergent validity, *r* = 0.89 [49] and reliability, *α* = 0.94–0.96 [42].

### Secondary outcome measures

As a secondary target criterion, the decrease in depression is recorded by the MADRS [47] and the second edition of BDI-II [43].

The MADRS [47] is a measure of change in depression. It contains 10 items which are scored from 0 to 6. Each item has its specific meaning for the rating, e.g. the item ‘Inability to feel’ is scored from 0 = “Normal interest in the surroundings and other people” to 6 = “The experience of being emotionally paralyzed, inability to feel anger, grief or pleasure and a complete or even painful failure to feel for close relatives and friends”. Another example item is reduced appetite, scored from 0 = “Normal or increased appetite” to 6 = “Needs persuasion to eat at all”. The MADRS has shown good validity, *r* = 0.63 and reliability, *α* = 0.85 [50].

The BDI-II [43] is a widely used scale of depression symptoms. It contains 21 self-report items which are scored from 0 to 3, with 3 being the highest severity. Example items include Crying, scored from 0 = “I don’t cry more often than I used to” to 3 = “I feel like crying, but I can’t” and Worthlessness, scored from 0 = “I do not feel I am worthless” to 3 = “I feel utterly worthless”. The BDI-II has been evaluated as valid *d* = 0.80 and reliable, *α* = 0.89 [51].

Improvement in general functional level in the verum vs. sham group is measured by the GAF [44]. The GAF describes an individual’s general functioning on a broad spectrum of activities in 10 steps, which each contain 10%, meaning an individual can score the lowest functional level of 1–10% meaning a persistent danger to self or others, a persistent inability to care for yourself or performed a serious suicidal act. The highest achievable level, 91–100%, no symptoms present, excellent functioning in a wide range of activities as well as good social standing due to self-presentation and a good handling of life obstacles. The GAF has been found to be reliable, *r* = 0.54 to 0.66 [52] and showing good discriminant validity, *r* = 0.63 [53].

A decrease in impulsivity is recorded by the BIS-15 [45]. The BIS-15 is a measure used for the identification of impulsive acts. It contains 15 items scored from 1 = ”Rarely/Never” to 4 = “ Almost Always/Always”. Example items include “I say things without thinking” and “I don’t pay attention”. The BIS-15 has been found to be valid, *r* = 0.37 = and reliable *α* = 0.81 [54].

Changes in self-compassion will be assessed by the German short version of the SCS-D [46]. The SCS-D measures the ability to have compassion for oneself rather than self-judgement. It contains 12 items scored from 1 = “very rarely” to 5 = “very often”. Example items include statements like “When I feel down, I tend to feel like most other people are probably happier than I am” and “I am disapproving and judgmental about my own flaws and inadequacies”. The short form of the SCS has been shown to be reliable, *α* = 0.86 and valid, *r* > 0.55 for all subscales [55].

### Intertemporal decision-making task (delay discounting)

Changes in impulsivity will be assessed by a cognitive task (delay discounting).

Delay discounting as a representative characteristic of impulsive behavior is especially important in BPD, which is often described as an impulse control disorder [56]. Krause-Utz et al. [57] found that delay discounting might be considered a general feature of BPD, with individuals affected by it showing a higher degree of delay discounting even when controlling for comorbid ADHD. The researchers also concluded that the number of induced stressors did not influence the degree of delay discounting in BPD individuals. This means that the degree of delay discounting is likely not influenced by external factors, but generally present in affected individuals.

The delay discounting paradigm is a very common way of measuring impulsiveness and self-control. The variant we are going to use was developed by Kirby et al. [48] and has already been validated in numerous clinical populations (e.g. in addiction disorders [48]; depression [58]; and schizophrenia [59]). In the assignment, patients will be asked to make a series of decisions where they have to choose between a small reward on the same day or a larger reward that is paid out after a waiting period. The task will include 27 rounds with different amounts of money (11–85 €) and different waiting times (7–186 days). At the end of the experiment, one of these 27 rounds will be randomly selected and the patients will be paid 10% of the chosen amount of money. If the patients have opted for the immediate reward in the selected round, they will receive the money on the same day. If a decision is made in favor of the later reward, the money will either be sent by post or, if the time is still during the inpatient stay, the patients will receive the money from the ward staff on the specified day. Since the patients complete the task twice, they can earn between 2.20 and 17 €.

### Structural MRI

Structural MRI data will be analyzed using the FreeSurfer software (versions 7.3.2, [60]). Analysis and quality-control protocols of the ENIGMA consortium will applied (http://enigma.ini.usc.edu/protocols/imaging-protocols) covering the recon-all -all stream and cortical as well as subcortical segmentation based on the Desikan–Killiany atlas. Data will be visually inspected and statistically evaluated for outliers. After quality control, volumes of 54 subcortical and cortical regions (27 per hemisphere) will be extracted, in addition to the intracranial volume to correct for global brain volume in statistical models.

### Proposed sample size and power calculation

In two small studies that examined the efficacy of rTMS in the treatment of borderline personality disorder, medium to large effect sizes were demonstrated (Cohen’s d = 0.74–2.79, [18, 61]). Since these pilot studies had small sample sizes, the possibility of an alpha error and an overestimation of the effect size cannot be ruled out. In order to use a conservative estimate, Whitehead et al. [62] recommend a group size of N = 15 for pilot studies if mean effect sizes are to be expected in the outcome parameter. Since we conduct a pilot study with an inter-subject design with two groups and allow for a 25% contingency for dropouts, we will include N = 53 participants in the study totally for both interventions. Based on a power/sample size calculation with G-POWER [63], the resulting total sample size of 40 patients is sufficient to detect a small to medium effect (f = 0.2) with alpha = 0.05 and a power (1-beta) of 80% (for testing the interaction in a repeated measurement design with two groups). Thus, with the projected sample size a clinical relevant effect of rTMS (in addition to DBT) can be revealed with sufficient power.

### Data analysis

Regarding therapy response, a 2 × 2-factorial between-subjects design with the between-subject factor stimulation (TMS vs. Sham) and the within-subject factor time (T0 vs. T1) will be conducted. To deal with a possible bias by treatment drop-out, a Linear Mixed Repeated Measure Model approach will be applied considering all assessed timepoints and the primary outcome (differences in BSL-23 reduction over time between study groups, i.e. interaction time*group) will be tested for significance (p ≤ 0.05). In case of relevant pre-treatment differences, these will be included in the model as covariates to adjust for. Likewise, secondary outcomes will analyzed accordingly.

In a second step, patients will be grouped into responder and non-responder. Group assignment will be performed post-hoc and based on the Reliable Change Index (RCI) as suggested by Jacobson and Truax [64]. An RCI above the 95% confidence limits (+/= 1.96) indicates a reliable change (p < 0.05). To identify parameters which are relevant for therapy response, group comparisons will be performed on secondary outcome variables of T0, using MANOVA models with the independent factor therapy response (responder vs. non-responder) and secondary outcome variables of T0 as dependent variables. Analyses will be corrected for multiple comparisons using Bonferroni Correction and partial eta-square will be determined to report effect size.

Analyses will be conducted with IBM SPSS [65].

## Discussion

BPD is a psychiatric disorder that leads to severe impairments in social interaction, self-harming and reduced quality of life. Several studies have shown neural correlates underlying symptom reduction after evidence-based psychotherapy treatment in patients with BPD [7–9]. Patients with BPD can experience distinct symptom alleviation from DBT, especially in terms of emotion regulation. Psychotherapeutic treatment of patients with BPD is characterized by a long treatment duration over several weeks [7]. The symptom pattern of patients with BPD (especially impulsivity) makes such a long treatment more difficult, in particular regarding acute fluctuations in symptom severity. Therefore, a treatment is necessary that facilitates these processes. While most rTMS protocols have a duration between 20 and 30 min, which, due to BPD symptoms, might complicate the recruiting of participants and enhance dropout rates, the iTBS protocol is both a safe and effective brain stimulation technique as well as economic and short. It therefore might adequately deal with the impulsivity aspect of patients with BPD and thus prevent dropout. A lesser dropout rate and a higher compliance can therefore be expected due to this decision. The limitation of the design being single blind instead of double blind is countered with surveying the patients' own assessment whether they are in the verum or sham condition, which is taking place after the first and the last iTBS session.

Brain stimulation techniques can possibly support the neural correlates of psychotherapeutic approaches. These techniques are well established in the treatment of other psychiatric disorders, especially depression [11]. In addition to that, first studies have shown that brain stimulation techniques can support symptom reduction in patients with BPD [15]. However, to our knowledge the combination of brain stimulation and evidence-based psychotherapy has not been examined to date. Our study aims to close this gap. For a first exploration of this research question, the manualized nature and regulated treatment duration of in-patient DBT makes it an appropriate choice for a first combination of iTBS and an evidence-based psychotherapy.

Due to the exploratory nature of our research question, the design will be conducted as a pilot study. It will provide evidence for feasibility and effect-size measures that allow for a data-based decision on the implementation, design and sample-size calculation of a confirmative multi-center trial. Our results might deliver answers to the questions if and how future studies should further investigate an enhancing effect of iTBS as an add-on to DBT or any other evidence-based psychotherapy in the treatment of BPD patients. Besides, we can identify necessary precautions and additional steps that can be taken to enhance the quality of further investigations. Furthermore, our study serves as an important addition to prospective investigations of DBT in a clinical setting itself.
